# Microfluidic flow switching *via* localized acoustic streaming controlled by surface acoustic waves†

**DOI:** 10.1039/c7ra11194k

**Published:** 2018-01-16

**Authors:** Jin Ho Jung, Ghulam Destgeer, Jinsoo Park, Husnain Ahmed, Kwangseok Park, Hyung Jin Sung

**Affiliations:** Department of Mechanical Engineering, KAIST Daejeon 34141 Korea hjsung@kaist.ac.kr

## Abstract

We propose an acoustic flow switching device that utilizes high-frequency surface acoustic waves (SAWs) produced by a slanted-finger interdigitated transducer. As the acoustic field induced by the SAWs was attenuated in the fluid, it produced an acoustic streaming flow in the form of a pair of symmetrical microvortices, which induced flow switching between two fluid streams in a controlled manner. The microfluidic device was composed of a piezoelectric substrate attached to a polydimethylsiloxane (PDMS) microchannel having an H-shaped junction that connected two fluid streams in the middle. The two immiscible fluids, separated by the PDMS wall, flowed in parallel, briefly came in contact at the junction, and separated again into the downstream microchannels. The acoustic streaming flow induced by the SAWs rotated the fluid streams within the microchannel cross-section, thereby altering the respective positions of the two fluids and directing them into the opposite flow paths. The characteristics of the flow switching mechanism were investigated by tuning the input voltage and the flowrates. On-demand acoustic flow switching was successfully achieved without additional moving parts inside the microchannel. This technique may be useful for fundamental studies that integrate complex experimental platforms into a single chip.

## Abstract 2

Acoustic streaming flow induced by high-frequency surface acoustic waves has been used to switch streams of two immiscible fluids flowing in parallel through a bifurcating microchannel with an H-shaped junction at the centre.

## Graphical abstract

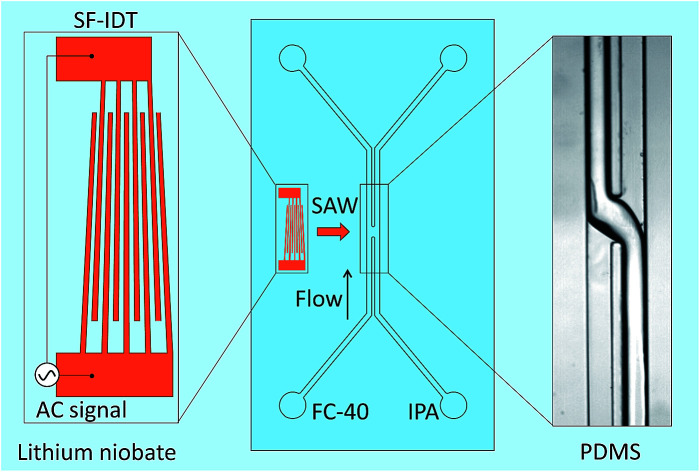

## Introduction

Miniaturizing benchtop experimental setups in palm-size microfluidic platforms has been the focus of recent research in micro-total analysis systems (μTAS).^1–4^ The development and integration of fundamental flow control modules, such as pumpless flow actuation and flow-directing valves, in microfluidic devices remains challenging due to a lack of inter-module compatibility.^5–9^ On-demand control over the fluid flow direction in a microfluidic channel is essential to the design of complex integrated experimental μTAS platforms that simultaneously perform distinct chemical reactions. The realization of all-encompassing microfluidic platforms capable of handling multiple biochemical assays on a single chip using polydimethylsiloxane (PDMS)-based pneumatic valves, which harness the elastic properties of the polymer material, has been difficult.^2,10,11^ Multiple PDMS layers form overlaying fluidic channels separated by a thin PDMS membrane that can be easily deformed to block liquid passage in one channel by applying an external air pressure to another channel. By integrating an additional air pressure control layer, complex flow networks may be built to enable fluid flow switching operation. The design and fabrication, with reasonable repeatability, of multi-layered microchannels in large quantities is difficult because the deformation rate of a PDMS wall depends strongly on the material elasticity, which cannot be readily controlled. The need for additional pneumatic pumps, which are typically larger than the microfluidic system, renders the experimental setup bulky and resistant to miniaturization.

Previous studies integrated a bubble gate into a single-layered PDMS microchannel to directly block a specific liquid flow path using an air bubble that did not require stacks of multiple PDMS layers.^9^ However, the liquid and gas pressures needed to be controlled individually, which meant that an additional pressurized module was required. Flow switching experiments using external forces have been introduced to build fluidic networks on a microchip. A thermocapillary force^12^ and surface acoustic waves (SAWs)^13^ were used in segmented flow control to switch dispersed liquid plugs in a microchannel. These methods were easily integrated into PDMS microchips; however, these and other efforts have not yet addressed the need to control the direction of a continuous flow in a microchannel.

Acoustofluidic miniaturized devices using SAW-based actuation were recently investigated for the dexterous handling of suspended micro-objects^14–18^ and fluids at the microscale.^3,19–25^ The use of an acoustic streaming flow produced by high-frequency SAWs is one of the most efficient methods of inducing localized high-speed fluid motions using two symmetric microvortices inside a microchannel.^26,27^ SAWs turn into a compressional leaky wave as they interact with the fluid medium in the microchannel positioned atop the piezoelectric substrate. The SAWs refract at the substrate surface as they radiate energy into the fluid, and the leaky SAWs are attenuated during propagation through the fluid medium. The acoustic waves' attenuation produces a pressure gradient in the direction of wave propagation that generates a time-averaged body force (*F*_B_) on the fluid, thereby producing a streaming flow in the form of micro-vortices. Microvortices produced by SAWs have been widely used in microfluidic platforms to mix fluids,^26^ sort particles,^27,28^ manipulate cells,^29,30^ drive SAW propulsion devices,^31,32^ manipulate droplets,^33–35^ and pump fluids using acoustic flows.^36^

In this study, we took advantage of localized micro-vortices to realize a flow switching system that could interchangeably direct the flows of two immiscible fluids, such as co-flowing aqueous and non-aqueous phases in parallel streams, into two separate outlet ports. The acoustic streaming effect, which has been used previously to drive segmented flows, was utilized here to uniquely control the fluid–fluid interface in a continuous fashion. Previously, we used a slanted-finger interdigitated transducer (SF-IDT) with an effective aperture size that was comparable to the microchannel width to generate a very narrow beam (∼10^2^ μm) of acoustic waves that deformed the fluid–fluid surface and split a droplet into two.^37^ A narrow acoustic wave beam was essential for achieving flow switching between two immiscible fluids at the microchannel junction, in which the fluids contacted one another before being separated by a thin PDMS wall downstream. We categorized four different flow-switching regimes during fluid–fluid interface actuation by acoustic waves of various amplitudes. The experimental observations and the mechanism underlying the flow switching behaviour could be explained in terms of the strong acoustic streaming flow generated at the microchannel junction.

## Experimental section

**Fig. 1:**
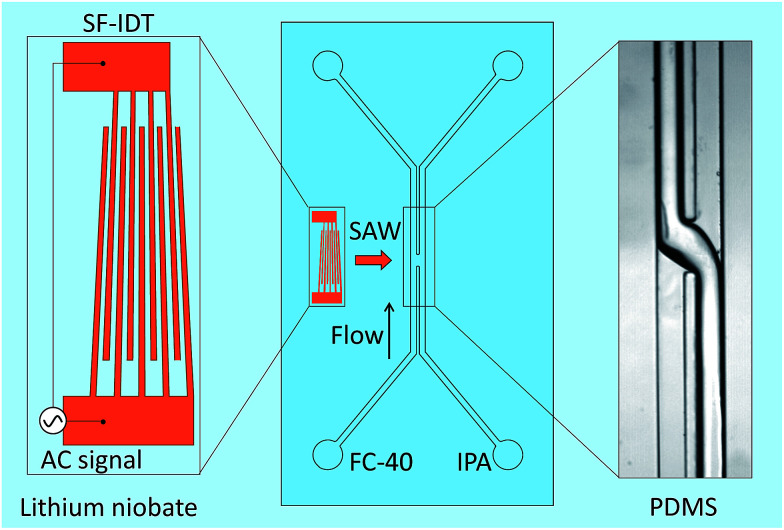
Schematic diagram showing the acoustic flow switching device comprising a polydimethylsiloxane (PDMS) microchannel and a slanted-finger interdigitated transducer (SF-IDT). The SF-IDT produced a narrow beam of surface acoustic waves (SAWs) directed at the small region of the fluid–fluid interface. FC-40 and isopropyl alcohol (IPA) flowed in parallel and briefly came into contact at the microchannel junction. Upon exposure to the SAWs, a strong acoustic streaming flow switched the directions of the FC-40 and IPA and induced the fluids to flow into different outlet ports separated by a thin PDMS wall.

A schematic diagram of the acoustic flow switching system is shown in Fig. 1. The device was composed of a PDMS microchannel attached to a piezoelectric substrate (lithium niobate (LN), LiNbO_3_, 128° Y-cut, MTI Korea) patterned with bimetallic interdigitated electrode finger pairs (Cr/Au, 300 Å/1000 Å, E-beam evaporation process). The bonding between the PDMS channel and the LN substrate was enhanced by coating the LN substrate with a SiO_2_ layer. The number of finger pairs was 40, and the SF-IDT aperture was 1 mm. The pitch of the finger pairs (*λ*) ranged from 28 to 36 μm, which corresponded to SAW frequencies (*f*_SAW_ = *c*_s_/*λ*) between 109 and 141 MHz. The effective aperture of the SF-IDT could be estimated as^38^1
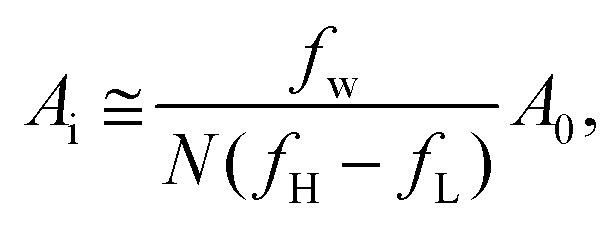
where *f*_w_ is the working frequency, *N* is the number of SF-IDT fingers, *f*_H_ and *f*_L_ are the highest and lowest frequencies from the SF-IDT, and *A*_0_ is the total aperture. The effective aperture of the SF-IDT was around 85 μm, smaller than the fluid–fluid interface at the junction. An AC electrical signal was provided by an RF signal generator (N5171B, Keysight Technologies), and the signal was amplified by an amplifier (UP-3015, Unicorn Tech.). The SAW voltage was measured using an oscilloscope (DSO-X 2022A, Keysight Technologies).

The PDMS microchannel was fabricated using soft lithography techniques and was attached to the LN substrate by oxygen plasma bonding. The microchannel width and height were 300 and 130 μm, respectively. The microchannel was composed of two inlets and two outlets, and the two fluids were separated by a 50 μm thick PDMS wall. In the acoustic actuation region at the microchannel junction, the wall was removed so that the SAWs could generate an acoustic streaming flow over the fluid–fluid interface to flip the fluid streams. The width of the open area at the junction of the fluid–fluid interface was 200 μm.

Isopropyl alcohol (IPA, Sigma Aldrich) and FC-40 (3M) were used as two immiscible fluids. A syringe pump (neMESYS, CETONI GmbH) with four independent units was used to control the flow rates of the two incoming fluids *via* pumping and the two outgoing fluids *via* flow suction. Suction of the outgoing fluid was important for stabilizing the fluid flow in the microchannel, especially at the junction. Experimental images were recorded using a high-speed camera (pco.1200 hs PCO camera) attached to an inverted microscope (Olympus IX71).

The interfacial tension between the two fluids was measured with the pendant drop method using a tensiometer (Biolin Scientific). The FC-40 pendant drop was formed at the tip of a stainless steel needle (OD = 0.72 mm) immersed in the IPA solution to enable drop-shape analysis. The interfacial tension was measured as *γ* = 5.2 mN m^−1^ that prevented segmented droplet production during flow switching at the microchannel junction.

## Results and discussion

**Fig. 2:**
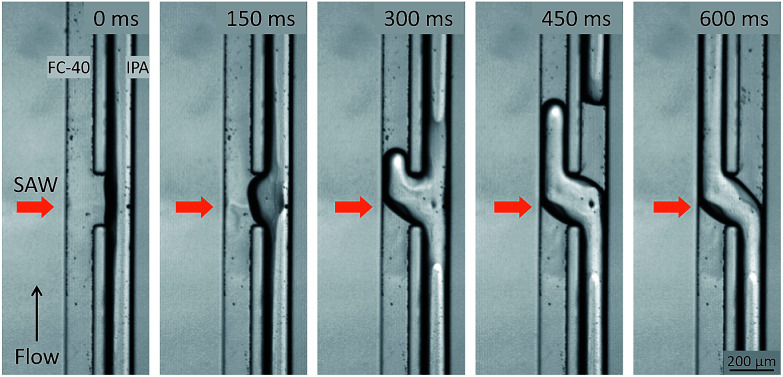
Experimental images of the acoustic flow switching actuated by the SAWs at a frequency (*f*_SAW_) of 109 MHz and a SAW voltage (*V*_SAW_) of 9.36 V_pp_. The flow rates of the aqueous and non-aqueous phase fluids were 200 μL h^−1^. The SAWs radiated within the microchannel toward the fluid–fluid interface and generated a strong acoustic streaming flow that gradually rotated the fluid streams and switched their directions within a second of device actuation.

Experimental images of the acoustic flow switching action are shown in Fig. 2. The IPA and FC-40 fluids flowed in parallel from the bottom to the top of the frame. The flow rate of both fluids was 200 μL h^−1^ each. The SAWs were directed toward the fluid–fluid interface. As the SAWs propagated into the microchannel and interacted with the fluid–fluid interface, the free surface began to deform between *t* = 0–150 ms. The recorded images were captured using an inverted microscope focused at the bottom of the microchannel to reveal changes in the interface geometry. The IPA (right) fluid was displaced toward the bottom-left of the microchannel cross-section (looking in the direction of flow) (*t* = 150 ms). At 300 ms, the IPA stream penetrated the left microchannel and replaced some of the volumetric flow of the FC-40 fluid, and a similar portion of the FC-40 moved toward the right microchannel. As the IPA stream blocked the left flow path and the FC-40 stream was deflected into the right side, the IPA stream inside the right channel broke entirely (at 450 ms), and the positions of the two fluids were completely altered (at 600 ms). The IPA and FC-40 streams were switched as they crossed each other at the microchannel junction when exposed to the SAW.

**Fig. 3:**
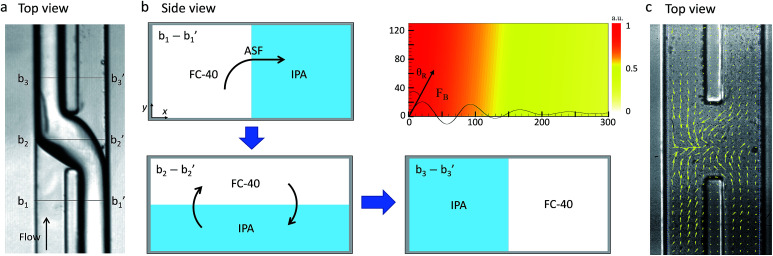
(a) Experimental image showing the acoustic flow switching. The lines b_1_-b_1_′, b_2_-b_2_′, and b_3_-b_3_′ indicate the positions corresponding to the schematic diagrams of the microchannel cross-sections show in (b). (b) Schematic diagram of the fluid interface displacement at the cross-section of the microchannel. Before the fluid streams met the acoustic field, the FC-40 stream and the IPA stream were positioned on the left and right sides, respectively, of the microchannel. At the center of the SAW beam, the FC-40 and IPA streams were rotated and positioned at the top and bottom, respectively, in the microchannel. The fluid stream continued to rotate in the microchannel cross-section until the FC-40 and IPA stream positions were switched (line b_3_-b_3_′). The mechanism by which the fluid streams rotated at the microchannel cross-section was investigated. The time-averaged body force due to the acoustic streaming effect is plotted in (b). The direction of the acoustic body force was equal to the Rayleigh angle at which the SAWs penetrated the fluid medium. (c) The velocity field image measured from the PIV experiment. The bulk velocity of the micro channel was stopped for measuring the velocity field induced by the acoustic field only. For the measurement, 1 μm polymer particles were used while the SAW voltage was 4.2 V_pp_ and the images were taken at 2500 fps. The maximum acoustic streaming velocity was measured as 1.13 mm s^−1^.

The mechanism underlying the acoustic flow switching phenomenon was investigated by characterizing the fluid stream cross-sectional profile at the fluid crossover position. Fig. 3 shows an experimental image (a, top view) and a schematic diagram (b, side view) of the acoustic flow switching. The experimental conditions were same as those described in Fig. 2. The FC-40 and IPA fluids flowed in parallel from the bottom to the top of the image frame prior to encountering the acoustic field at the microchannel junction. The experimental image (a, top view) was collected using an inverted microscope. The IPA stream was positioned at the bottom of the microchannel in the b_2_-b_2_′ cross-sectional diagram. The schematic diagrams shown in cross-sectional view in Fig. 3(b) illustrate the flow switching mechanism. The IPA and FC-40 fluids were parallel in the microchannel in the b_1_-b_1_′ cross-section. As the fluid streams entered the SAW actuation region, the IPA stream bent toward the left flow path while the FC-40 stream entered the right flow path (b_2_-b_2_′). The experimental results suggested that the fluid streams rotated in the clockwise direction within the microchannel cross-section which is consistent with the direction of the acoustic streaming flow field produced by a SAW.^39^ As the fluid streams flowed from the b_2_-b_2_′ to the b_3_-b_3_′ configurations, the IPA and FC-40 streams flowed into the left and right flow paths, respectively. The two fluid streams were completely altered and clearly separated from one another. Their trajectories remained stable after cross-over. These results suggested that the fluid streams rotated continuously in the clockwise direction until they passed the SAW actuation region. The b_3_-b_3_′ cross-section shows that the two fluids were again separated by the thin PDMS wall, and no additional fluid stream translation occurred.

The clockwise direction of the fluid stream rotation could be understood in terms of the acoustic streaming effect. When the SAWs met the fluid medium inside the microchannel, the SAWs turned into a leaky wave that propagated at the Rayleigh angle *θ*_R_ from the surface normal direction, described according to2
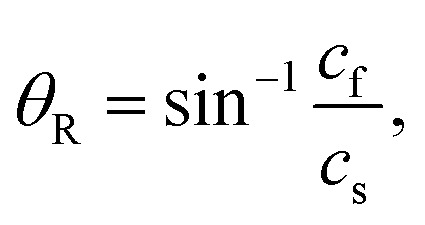
where *c*_f_ and *c*_s_ are the sound speeds in the fluid medium and the substrate, respectively. The typical SAW frequencies used in the microchannel ranged from MHz to GHz. The high-frequency SAWs quickly attenuated in the fluid medium as they propagated, while the amount of energy transferred to the fluid medium was proportional to the SAW frequency. The high spatial gradient of the acoustic field intensity was required for direct acoustic streaming generation. Thus, the acoustic streaming was easily generated when the acoustic field was highly localized and the SAW wavelength was small. The acoustic gradient was generated using focused interdigitated transducers (FIDT)^27^ or SF-IDT.

The time-averaged body force within a microchannel, induced by acoustic streaming, has been thoroughly studied.^27,40^ The body force in the direction of the Rayleigh wave could be expressed as3
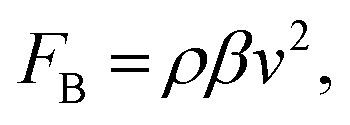
where *ρ* is the liquid density, *β* is the attenuation coefficient in the fluid, and *v* is the displacement velocity. The attenuation coefficient between the piezoelectric surface–fluid interface (*α*) and the fluid medium (*β*) could be expressed as^41^4
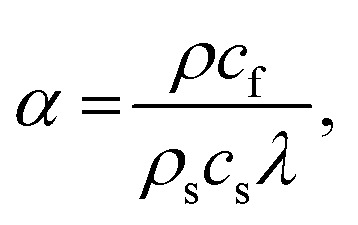
5
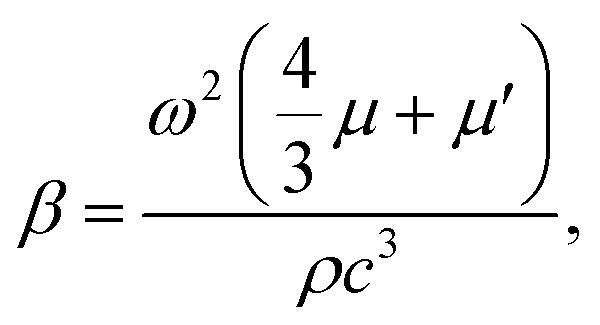
where *λ* is the SAW wavelength at the LN substrate, and *ω* is the corresponding angular frequency. *μ* and *μ*′ are the dynamic and bulk viscosities of the fluid medium. The inverse values of the attenuation coefficients were the characteristic length of the SAW propagation, such that higher attenuation coefficients corresponded to larger acoustic streaming. Collins *et al.* evaluated the particle displacement magnitude induced by the SAWs by considering the reflections at the microchannel boundaries.^27^ The first reflection at the microchannel roof was considered to estimate the particle velocity according to6
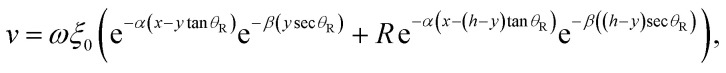
7
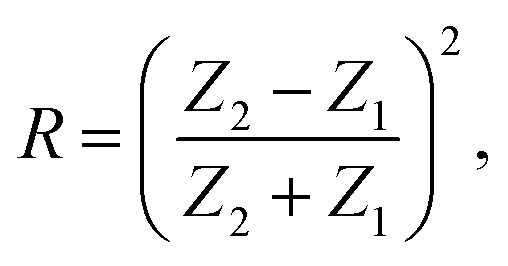
where *ξ*_0_ is the magnitude of the particle displacement at the left corner of the microchannel, *h* is the microchannel height, *R* is the acoustic wave reflection coefficient, and *Z*_1_ and *Z*_2_ are the acoustic impedance values of the fluid medium and PDMS, respectively. The acoustic streaming trends were observed by assuming that the fluid medium was FC-40.

The time-averaged body force distribution due to the acoustic streaming effects is shown in the upper right corner in Fig. 3(b). The horizontal and vertical axes of the graphs represent the lateral width and vertical height of the microchannel. The magnitude of the body force was normalized to 1. The direction of the acoustic body force was aligned with the Rayleigh angle at which the SAWs penetrated the fluid medium. The body force induced by the acoustic streaming was stronger at the bottom-left corner of the microchannel. The body force decreased as the SAWs propagated along the fluid medium, inducing flow displacement in the clockwise direction within the microchannel cross-section. The fluid velocity was stronger on the left side of the channel and weaker on the right side. The fluid streams can be easily displaced; however, to induce a complete switching of the fluid streams, it is essential to rotate them 180° in the clockwise direction.

**Fig. 4:**
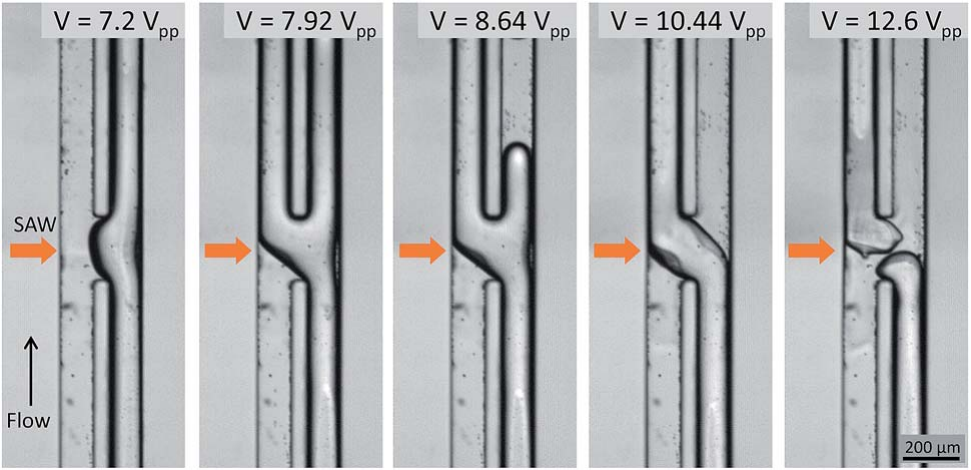
Experimental images of the acoustic flow switching as a function of the SAW voltage. The flow rates of the IPA and FC-40 stream were equal and set to 200 μL h^−1^ each while the SAW voltage ranged from 7.2 to 12.6 V_pp_. The experimental results revealed four distinct acoustic flow switching regimes. At 7.2 V_pp_, the acoustic streaming effect was not sufficiently strong to push the fluid streams into the opposite channels; however, the IPA stream (right side) assumed a concave shape at the SAW actuation region. As the SAW voltage increased, the IPA stream partially flowed into the left flow path (*V*_SAW_ = 7.92 and 8.64 V_pp_.) Stable acoustic flow switching was achieved when the SAW voltage was increased to 10.44 V_pp_. In this regime, the IPA and FC-40 fluid streams crossed one another at the microchannel junction under the effects of the SAW beam and the acoustic streaming effect. At higher SAW voltages (*V*_SAW_ = 12.6 V_pp_), the acoustic flow switching was still achieved; however, the flow streams sporadically broke under the much stronger streaming flow micro-vortices in the *xz* plane. An experimental video illustrating acoustic flow switching by varying the SAW voltage is provided in the ESI I.†

**Fig. 5:**
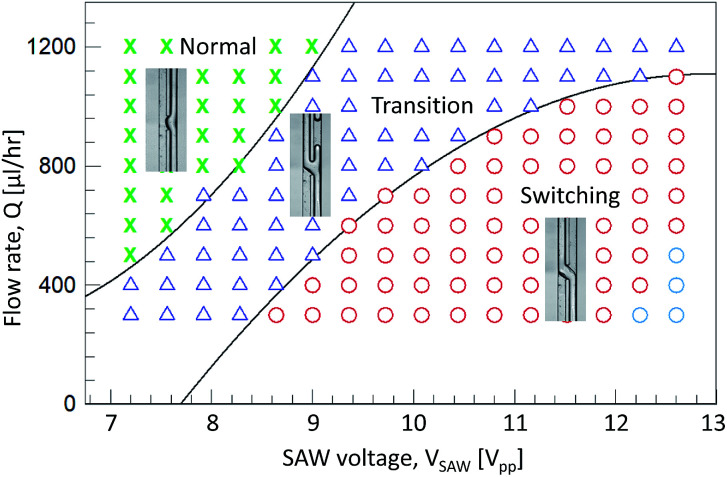
A regime diagram of the acoustic flow switching by controlling the flow rate and the SAW voltage. The symbols ‘x’, ‘Δ’, and ‘o’ represent the normal, transition, switching regime, respectively. The blue symbol ‘o’ indicates the unstable flow switching regime. The transition lines between the each regime were roughly estimated.

In order to verify the acoustic streaming flow effect, a PIV experiment was conducted by using 1 μm diameter polymer particles suspended in the IPA solution. The SAW voltage was set to a lower value of 4.2 V_pp_ to generate a low velocity streaming flow due to the image acquisition limitation (2500 fps) while the bulk fluid flow was stopped. The velocity field produced by the SAW is shown in Fig. 3(c). The maximum velocity of the micro vortex was measured as 1.13 mm s^−1^. As the acoustic streaming flow velocity is proportional to the square of the SAW voltage, we estimated the maximum streaming flow velocity of this system to be 3.32 to 10.16 mm s^−1^ corresponding to the input voltages of 7.2 to 12.6 V_pp_ (Fig. 4). The bulk fluid flow rate used in the system ranged from 300 to 1200 μL h^−1^ which corresponds to the mean flow velocity of 2.1 to 8.5 mm s^−1^. For a given flowrate, the input voltage is adjusted to induce a sufficiently strong acoustic streaming flow velocity to rotate the fluid streams at the micro channel cross-section before the fluid flowed past the SAW actuation zone. For example, when a net flowrate of 800 μL h^−1^ (5.68 mm s^−1^) of the two co-flowing streams of immiscible fluids was interrupted by streaming flow with a maximum velocity of 3.32 mm s^−1^ at 7.2 V_pp_ input voltage, the streaming velocity was not strong enough to induce a switching flow regime (see Fig. 5). As the input voltage was increased to 9.0 V_pp_, the maximum streaming velocity of 5.20 mm s^−1^ approached the average bulk velocity of 5.68 mm s^−1^, which resulted in a transition regime. However, the switching regime was only realized when the input voltage was further increased to a higher value of 10.44 V_pp_ resulting in a maximum streaming velocity of 7.00 mm s^−1^ greater than 5.68 mm s^−1^.

The experimental acoustic flow switching results obtained by varying the SAW voltage are shown in Fig. 4. The flow rates of the IPA and FC-40 streams were equal, 200 μL h^−1^, and the SAW voltage ranged from 7.2 to 12.6 V_pp_. When the SAW voltage was low (*V*_SAW_ = 7.2 V_pp_), the acoustic streaming was insufficiently strong to rotate the fluid stream at the microchannel cross-section. As a result, the IPA stream deformed to form a convex shape in the SAW actuation region. As the SAW voltage increased (*V*_SAW_ = 7.92 and 8.64 V_pp_), the IPA stream began to deflect toward the left flow path; however, the acoustic streaming velocity was insufficient to displace the whole IPA stream to the left side, and the IPA stream was divided among both flow paths. The IPA stream was not fully rotated in the microchannel cross-section (see Fig. 3(b)) before the fluid stream passed the acoustic actuation zone. In this regime (transition regime), each fluid stream was split amongst the two flow paths. A SAW voltage of 10.44 V_pp_ fully switched the IPA and FC-40 fluid streams to the opposite flow paths. Within the SAW working region in the microchannel cross-sectional area, the fluid streams were rotated 180°. The flow switching effect remained active when the power was on, and the fluid stream restored its flow direction as the power was turned off. An input voltage of 12.6 V_pp_ rendered the fluid stream unstable, although the acoustic flow switching effect remained valid. The horizontal microvortex was strong enough to break the fluid stream, and the IPA stream continued to break and reconnect. In this regime, the acoustic flow switching worked normally, but the excessive acoustic energy could break the fluid stream or make satellite droplets. A video collected from an experimental study is provided in the ESI I.†

The acoustic flow-switching trend was investigated by generating a regime diagram, as shown in Fig. 5, by controlling the flow rates and SAW powers. The net flow rate ranged from 300 to 1200 μL h^−1^, and the SAW voltage ranged from 7.2 to 12.6 V_pp_. During the experiment, the two inlet and two outlet flow rates remained unchanged. The outlet flow rates were controlled by applying a negative pressure using the syringe pump. The symbols ‘x’, ‘Δ’, and ‘o’ represent the normal, transition, and switching regimes, respectively. The sky blue symbol ‘o’ indicates the unstable flow-switching regime. In the normal regime, the streams did not cross one another, and only displayed a concave shape in the SAW actuation region (Fig. 4. *V*_SAW_ = 7.2 V_pp_). In this regime, the displaced fluid streams in the microchannel cross-sectional image were restored until the flow passed the SAW working zone. In the transition regime, the two fluid streams were divided into the two flow path and formed a Y-shaped flow pattern. As the SAW voltage increased, IPA stream volume that switched to the left flow path increased (see Fig. 4, case *V*_SAW_ = 7.92 and 8.64 V_pp_, ESI I†). In the switching regime, the fluid streams stably crossed to follow the opposite flow path. The transition flow lines were estimated using second-order polynomials (normal-transition line: *Q* = 17.2*V*_SAW_^2^ − 371.5*V*_SAW_ + 2239.2, transition-switching line: *Q* = −10.2*V*_SAW_^2^ − 527.1*V*_SAW_ − 5691.7).

**Fig. 6:**
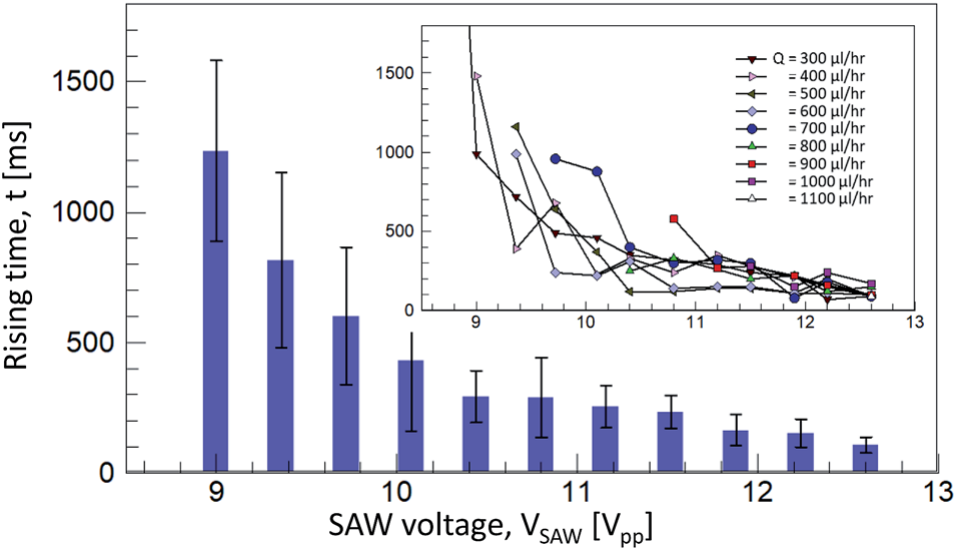
A rising time measurement of the acoustic flow switching in the switching regime in Fig. 5. The inset figure shows the rising time of each case while the flow rates and the SAW voltages were varied. The horizontal and vertical axes are same as in this figure. The graph shows that the rising time is highly dependent on the SAW voltage not the flow rate. The vertical bars in the main graph show the average rising time for the each flow rates. The error bars are the standard deviation of the measurement.

The acoustic flow-switching rise time was measured as shown in Fig. 6. The flow switching speed was determined by the flow switching rising time. At the moment of flow switching, the IPA fluid stream disconnected from its original fluid stream and bent toward the left flow path. The rising time was defined as the time delay between the moments of the SAW operation tuned ON to the disconnection of the IPA fluid stream. The bars and error bars indicate the average and standard deviation of the rising time. The inset figure shows the rising time measured at various flow rates and SAW voltages. The rising time decreased as the SAW voltage increased. The flow rate was not clearly correlated with the rising time, as shown in the inset. These results indicated that the flow switching speed depended solely on the acoustic streaming speed, although the feasibility of flow switching was a function of the acoustic streaming and the flow rate. The average rising time of the flow switching raged from 108 to 1235 ms. In the acoustofluidic device, the rising time was limited to 100 ms, and the value may decrease as the microchannel geometry and SAW frequency are tuned.

## Conclusions

In summary, we demonstrated an acoustic flow switching device that altered parallel flow streams using the acoustic streaming effect. The high-frequency localized SAWs in the fluid medium directly generated a microvortex inside the microchannel. The microchannel used in the device included two parallel flow paths and an open window (junction) that enabled SAW interaction with the fluid–fluid interface. At the SAW actuation region, the acoustic streaming rotated the fluid stream in the microchannel cross-sectional view. As a result, the fluid streams were rotated by 180° to alter the positions, and the streams completely crossed to follow the opposite flow path. The mechanism underlying the acoustic flow switching was investigated by considering the acoustic streaming theory in the microchannel. The characteristics of the acoustic flow switching were investigated by tuning the SAW voltage and flow rate. The flow switching phenomena were categorized as the flow switching, transition, and normal regimes. In the normal regime, the acoustic streaming deflected the fluid stream shape along a convex arc in the SAW working zone. In the transition regime, the fluid streams were partially switched and were divided among the two flow paths. On the other hand, in the switching regime, the fluid streams were completely crossed in the microchannel cross-section, and their positions were stably switched within one second. The rising time of the acoustic flow switching was measured as a device characteristic. The results revealed that the rising time depended on the acoustic streaming power, with a low correlation with the bulk fluid flow rate. The experimental results successfully demonstrated the flow switching operation without the use of additional moving parts in the microchannel. This technique may be useful for fundamental studies of microchannel applications in which complex experimental platforms are integrated into a single chip.

## Conflicts of interest

There are no conflicts to declare.

## Supplementary Material

RA-008-C7RA11194K-s001
